# Genome sequencing identifies somatic *BRAF* duplication c.1794_1796dupTAC;p.Thr599dup in pediatric patient with low-grade ganglioglioma

**DOI:** 10.1101/mcs.a002618

**Published:** 2018-04

**Authors:** Katherine E. Miller, Benjamin Kelly, James Fitch, Nicole Ross, Matthew R. Avenarius, Elizabeth Varga, Daniel C. Koboldt, Daniel R. Boué, Vincent Magrini, Scott L. Coven, Jonathan L. Finlay, Catherine E. Cottrell, Peter White, Julie M. Gastier-Foster, Richard K. Wilson, Jeffrey Leonard, Elaine R. Mardis

**Affiliations:** 1 Institute for Genomic Medicine, Nationwide Children's Hospital, Columbus, Ohio 43205, USA;; 2 Division of Hematology/Oncology/Bone Marrow Transplantation, Nationwide Children's Hospital, Columbus, Ohio 43205, USA;; 3 Department of Pediatrics, The Ohio State University College of Medicine, Columbus, Ohio 43210, USA;; 4 Department of Pathology and Laboratory Medicine, Nationwide Children's Hospital, Columbus, Ohio 43205, USA;; 5 Department of Neurosurgery, Nationwide Children's Hospital, Columbus, Ohio 43205, USA

**Keywords:** glioma

## Abstract

Gangliogliomas (WHO grade I) are rare tumors affecting the central nervous system and are most frequently observed in children. Next-generation sequencing of tumors is being utilized at an increasing rate in both research and clinical settings to characterize the genetic factors that drive tumorigenesis. Here, we report a rare *BRAF* somatic mutation (NM_004333.4:c.1794_1796dupTAC; p.Thr599dup) in the tumor genome from a pediatric patient in her late teens, who was initially diagnosed with low-grade ganglioglioma at age 13. This duplication of 3 nt introduces a second threonine residue at amino acid 599 of the BRAF protein. Based on previous studies, this variant is likely to increase kinase activity, similar to the well-characterized *BRAF* p.Val600Glu (V600E) pathogenic variant. In addition, although the p.T599dup somatic mutation has been documented rarely in human cancers, the variant has not been previously reported in ganglioglioma. The identification of this variant presents an opportunity to consider targeted therapy (e.g., BRAF inhibitor) for this patient.

## CASE PRESENTATION

A female patient, currently 18 yr of age, was diagnosed with a low-grade glioma at age 13 yr. She presented with blurred vision and difficulty seeing at distances. She was initially seen by an ophthalmologist who found swelling of the optic nerve and recommended an MRI of the brain, which revealed a midbrain mass extending to the medial left thalamus as well as obstructive hydrocephalus. The patient was subsequently admitted to the hospital for surgery and an endoscopic third ventriculostomy (ETV) was placed. Given the location and risk to critical structures, a biopsy of the mass was not obtained at that time; the location of the mass was consistent with a low-grade glioma. The patient did not display other symptoms prior to diagnosis (e.g., gait issues, headaches, motor weakness, vomiting, or seizures). Postoperative follow-up included a physical exam and MRI surveillance imaging every 3–4 months. During this time, imaging demonstrated slow interval growth, but the patient reported no clinical symptoms that were related to the tumor growth (e.g., headaches, vision changes). The patient was referred for chemotherapy but has not yet undergone any therapies. Four years after her initial diagnosis, a surveillance MRI indicated some central enhancement in the midbrain lesions that could represent a cyst; follow-up MRI demonstrated increased diffusion restriction and open biopsy was recommended. A needle biopsy confirmed a diagnosis of ganglioglioma, WHO grade I, and provided the specimen studied by next-generation sequencing (NGS) assays, as described herein.

## TECHNICAL ANALYSIS AND METHODS

### Whole-Genome and Whole-Exome Sequencing

Whole-exome sequencing (WES) and whole-genome sequencing (WGS) were performed on DNA isolated from the tumor biopsy and from peripheral blood (PBMC; i.e., normal comparator). Genomic DNA was processed for WGS using NEBNext Ultra II library prep. WES libraries were captured with the Agilent SureSelect v6 Exome kit (Agilent Technologies). Paired-end 151-bp reads were generated for exome-enriched and WGS libraries sequenced on the Illumina HiSeq 4000. Reads were aligned to the human genome reference sequence, build GRcH37, using Churchill and evaluated (Kelly et al. 2015). Sequence alignments were refined according to community-accepted guidelines for best practices (https://www.broadinstitute.org/gatk/guide/best-practices). Duplicate sequence reads were removed using samblaster-v.0.1.22, local realignment was performed on the aligned sequence data using the Genome Analysis Toolkit (GATK) (v3.7–0), and Churchill's own deterministic implementation of base quality score recalibration was used. The GATK's HaplotypeCaller was used to call germline variants. Average sequencing coverage depth for the tumor sample was 221× (WES) and 55× (WGS); for the normal sample, coverage was 230× (WES) and 31× (WGS). Somatic single-nucleotide variation (SNV) and indel detection was performed for the WGS and WES data sets separately using MuTect 2 (Cibulskis et al. 2013). Detection of copy-number variation (CNV), as well as somatic and germline structural variation (SV), was performed using WGS data.

### Sanger Sequencing

DNA derived from peripheral blood and tumor was independently used as template to PCR amplify *BRAF* exon 15 using the following primer sequences: exon 15F 5′-GTAAAACGACGGCCAGACTCTTCATAATGCTTGCTCTGA-3′ and exon 15R 5′-CAGGAAACAGCTATGACAGTAACTCAGCAGCATCTCAGG-3′. The resulting 251-bp products were purified (QIAGEN, 28106) and Sanger sequenced (ThermoFisher, 4336943) using M13 forward and reverse primers. Electropherograms were analyzed using Sequencher version 5.3.

### RNA-seq

DNase-treated, ribo-depleted total tumor RNA was used as input for library construction using Illumina's TruSeq Stranded Total RNA Sample prep. RNA-seq data were processed using STAR-Fusion, and these data were used to detect putatively expressed gene fusions in the tumor (https://github.com/STAR-Fusion). We analyzed 125,302,701 RNA-seq reads.

## VARIANT INTERPRETATION

SnpEff and custom in-house scripts were used to annotate variants (mutation and gene information), predict their functional impact on proteins, and assign population allele frequencies (Cingolani et al. 2012). Variants observed in the matched normal sample (i.e., germline) were excluded from somatic analysis. Common variants (MAF > 1%), variants outside of coding regions (>4 bp from an exon splice site), and exonic synonymous variants were also excluded from analysis. Potential variants were screened for cancer relevance based on previous reports in ClinVar, COSMIC, dbSNP, ICGC, and TCGA databases.

## RESULTS

**Figure 1.**
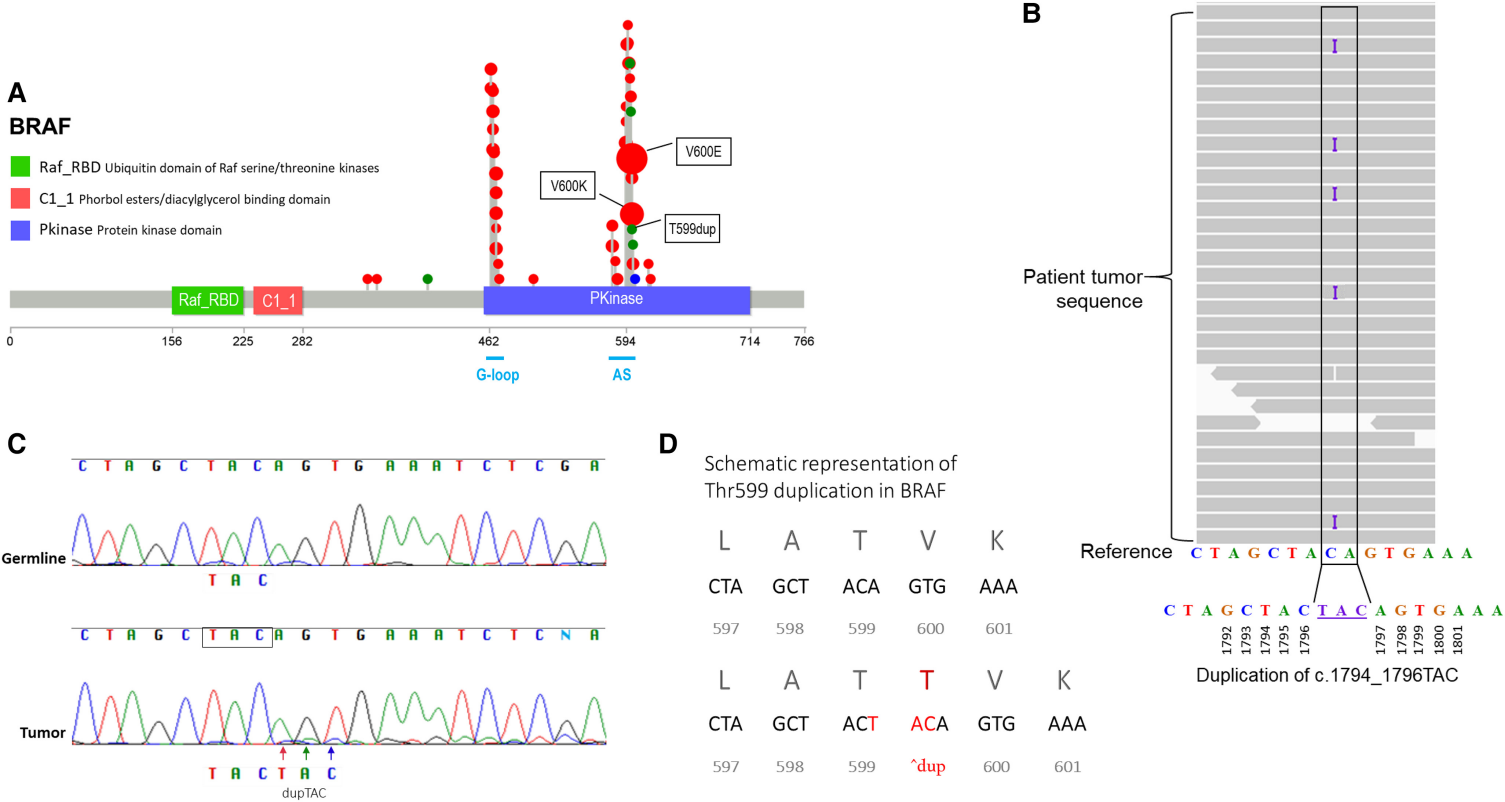
(*A*) *BRAF* mutations identified in tumor sequences. BRAF protein is represented here with annotated domains and amino acids (0–766) numbered underneath. Plots were generated (https://github.com/pbnjay/lollipops) with recurrent *BRAF* mutations (red, missense; green, indel; blue, nonsense) identified in COSMIC (Bamford et al. 2004), with larger dots (not to scale) indicating higher-frequency mutations. Most tumor-associated BRAF variants, including the well-characterized p.V600E and p.V600K and the less frequent p.T599dup variant we report, are within the protein kinase domain, specifically in the conserved glycine motif (G-loop) or in the activation segment (AS) in exon 11 and 15, respectively. (*B*) Integrated Genomics Viewer (IGV) display of the c.1794_1796dupTAC. Analysis of the tumor DNA revealed a variant allele frequency of ∼8.3% (variant allele present in 5/60 reads). (*C*) Sanger sequencing of *BRAF* exon 15 in germline (peripheral blood) and tumor. The arrows indicate the TAC duplication, observed at low frequency, only in the tumor sample. The low peaks seen after the duplication correspond to the offset bases, because of the 3-bp duplication. (*D*) Schematic representation of Thr599 duplication. The specific TAC duplication is an in-frame variant, which duplicates the threonine residue at amino acid 599.

After filtering as described above, a total of 15 somatic nonsynonymous variants were further evaluated for cancer relevance. Our somatic analysis detected a pathogenic variant c.1794_1796dupTAC;p.Thr599dup in the *BRAF* gene (Table 1). The duplication of three nucleotides results in an in-frame introduction of a threonine residue at amino acid 599 (Fig. 1). Histology reports indicated a 70% tumor cellularity; however, the variant allele frequency (VAF) of somatic mutations suggests a much lower actual tumor cellularity. To return the results to the physician for patient care, clinical confirmation of the variant was performed by amplification of exon 15 of the *BRAF* gene, followed by bidirectional Sanger sequencing. Our initial confirmation assay was negative, because of low tumor content in the sample and the relative insensitivity of Sanger assays. As such, we identified a second portion of the biopsy specimen with a higher predicted tumor cellularity and isolated DNA for the Sanger confirmation, which indicated the variant was present at a low, but detectable, allelic frequency (Fig. 1). In parallel, we identified the presence of this variant and expression of the variant allele in RNA-seq data. We discovered a gene fusion transcript *TFG-GPR128* in the RNA-seq data, which we determined to be irrelevant because this particular fusion has been identified in multiple healthy individuals (Chase et al. 2010). WGS analysis did not identify any LOH/CNV regions in the tumor. Other variants (SNVs, indels, SVs) involving cancer genes were not identified in our somatic analyses. Germline WES and WGS analysis did not reveal any pathogenic variants in known cancer predisposition genes (Zhang et al. 2015).

**Table 1.** Genome sequencing results

Gene	Chr	HGVS cDNA	HGVS protein	Allele origin	Predicted effect	Read depth of variant position	Variant allele frequency, tumor N reads (%)
*BRAF*	7q34	NM_004333.4: c.1794_1796dupTAC	p.Thr599dup	Somatic	Increased kinase activity (Eisenhardt et al. 2011)	WGS: 60× WES: 172×	WGS: 5/60 (8.3%) WES: 6/172 (3.5%)

HGVS, Human Genome Variation Society; WGS, whole-genome sequencing; WES, whole-exome sequencing

## SUMMARY

Previous in vitro studies of the p.T599dup variant demonstrated kinase activity and cellular MEK/ERK activation potential comparable to that of *BRAF* p.V600E, suggesting that a similar therapeutic approach as for V600E may be effective (Eisenhardt et al. 2011). Presumably, the duplication of the threonine residue destabilizes the inactive conformation of the kinase domain. The c.1794_1796TACdup variant was classified as Tier II, Level C variant in accordance with the AMP/ASCO Standards and Guidelines for the Interpretation and Reporting of Sequence Variants in Cancer (Li et al. 2017). The variant has a potential clinical actionability based on reports demonstrating therapeutic and diagnostic/prognostic utility of BRAF p.V600 substitution mutations. Although the c.1794_1796dupTAC somatic variant has been observed in other cancers, notably in thyroid cancer, melanoma, and pilocytic astrocytoma, it has not been previously described in low-grade ganglioglioma (Jones et al. 2009; Yu et al. 2009; Gauchotte et al. 2011; Menzies et al. 2012). Furthermore, the variant was not identified in germline DNA of more than 15,000 individuals in the gnomAD database (Lek et al. 2016).

We describe a somatic in-frame mutation in exon 15 of *BRAF* in the tumor genome of a pediatric patient with ganglioglioma. The mutation duplicates the threonine residue at amino acid 599. Somatic mutations of *BRAF* are found with particularly high frequency in melanoma and colorectal, ovarian, and thyroid carcinomas (Davies et al. 2002; Curtin et al. 2005; Murugan et al. 2016). Specifically, the p.V600E variant accounts for >70% of reported somatic pathogenic mutations in *BRAF* (Wan et al. 2004; Watson et al. 2013). Specific to gangliogliomas, BRAF substitutions are quite common. For example, BRAF p.V600E has been observed in 25%–35% of adult and pediatric gangliogliomas (Schindler et al. 2011; Qaddoumi et al. 2016), whereas other *BRAF* genetic alterations and *KIAA1549-BRAF* and *FAM131B-BRAF* fusions have also been observed in low-grade gliomas, the former fusion having been identified in gangliogliomas (Dimitriadis et al. 2013; Gupta et al. 2014; Roth et al. 2015). All of the above BRAF alterations cause increased activation of BRAF and therefore constitutive activation of the mitogen-activated protein kinase (MAPK) pathway. Previous studies have indicated that the c.1794_1796dupTAC mutation described here is pathogenic and mimics the increased oncogenic kinase activity associated with BRAF p.V600E (Eisenhardt et al. 2011). Inhibitors that can target aberrant BRAF expression (e.g., vemurafenib, dabrafenib) have been shown to positively impact disease outcomes and treatment responses in patients with BRAF V600 substitutions (Flaherty et al. 2012; Hyman et al. 2015; Karoulia et al. 2017). Specific therapeutic agents based on our patient's oncogenotype are currently being pursued.

Beyond tumor resection, radiation therapy, or chemotherapy, which often directly lead to comorbidities and/or adverse events, it is anticipated that this type of targeted therapy offers an alternative treatment strategy for this patient. It is exciting and encouraging to think about translating knowledge of somatic mutations into effective therapeutic agents, as the neuro-oncology community continues to reveal the molecular landscape of rare, pediatric brain tumors.

In conclusion, we suggest that screening for somatic *BRAF* alterations should extend beyond the well-documented hotspot variant BRAF p.V600E, as the pathogenic variant p.T599dup described here mimics the increased oncogenic kinase activity associated with p.V600E (Eisenhardt et al. 2011). It is critical that genetic assays, particularly for *BRAF* mutations, are capable of detecting a full range of genotypes, as the identification of pathogenic variants can lead to targeted treatment approaches for patients, as discussed here.

## ADDITIONAL INFORMATION

### Database Deposition and Access

The variant has been deposited in ClinVar (https://www.ncbi.nlm.nih.gov/clinvar/) under accession number SCV000611854.

### Ethics Statement

This study was reviewed and approved by the Institutional Review Board (IRB) of The Research Institute at Nationwide Children's Hospital. Consent was obtained from the patient and parents for molecular genetic workup, including whole-exome and whole-genome sequencing. The protocol allowed for return of results from research sequencing studies after confirmation in a CLIA-certified laboratory.

### Acknowledgments

We thank the patient and the family for their participation in our studies. We also thank the Nationwide Insurance Innovation Fund for their support of the Institute for Genomic Medicine.

### Author Contributions

K.E.M. contributed to variant interpretation, initial manuscript preparation, and revision; B.K. and J.F. contributed to bioinformatics and data processing; N.R. contributed to sample processing and evaluation; M.R.A. contributed to clinical variant confirmation; E.V. and S.L.C. contributed to gathering clinical history represented in the case presentation; D.C.K. contributed to bioinformatics and variant interpretation; D.R.B. contributed to pathology and diagnostic workup; V.M. contributed to study design; J.L.F. contributed to clinical care of patient; C.E.C. and J.M.G.-F. contributed to study design and variant interpretation; P.W. contributed to study design and bioinformatics; R.K.W. contributed to study design and initial manuscript preparation; J.L. contributed to clinical care and neurosurgery of patient; and E.R.M. contributed to study design, variant interpretation, initial manuscript preparation, and revision. All authors read and approved the final version of the manuscript.

### Funding

This work was supported by the Nationwide Children's Hospital Institute for Genomic Medicine strategic fund.

### Competing Interest Statement

The authors have declared no competing interest.
